# Mechanisms of Neuroplasticity and Ethanol’s Effects on Plasticity in the Striatum and Bed Nucleus of the Stria Terminalis

**DOI:** 10.35946/arcr.v37.1.08

**Published:** 2015

**Authors:** David M. Lovinger, Thomas L. Kash

**Affiliations:** David M. Lovinger, Ph.D., is a senior investigator in the Laboratory for Integrative Neuroscience, National Institute on Alcohol Abuse and Alcoholism, Bethesda, Maryland.; Thomas L. Kash, Ph.D., is an assistant professor at the Bowles Center for Alcohol Studies and in the Department of Pharmacology, University of North Carolina School of Medicine, Chapel Hill, North Carolina.

**Keywords:** Alcohol consumption, ethanol exposure, alcohol use disorder, relapse, brain, neuroplasticity, synaptic function, synaptic plasticity, striatum, stria terminalis, bed nucleus of the stria terminalis

## Abstract

Long-lasting changes in synaptic function (i.e., synaptic plasticity) have long been thought to contribute to information storage in the nervous system. Although synaptic plasticity mainly has adaptive functions that allow the organism to function in complex environments, it is now clear that certain events or exposure to various substances can produce plasticity that has negative consequences for organisms. Exposure to drugs of abuse, in particular ethanol, is a life experience that can activate or alter synaptic plasticity, often resulting in increased drug seeking and taking and in many cases addiction. Two brain regions subject to alcohol’s effects on synaptic plasticity are the striatum and bed nucleus of the stria terminalis (BNST), both of which have key roles in alcohol’s actions and control of intake. The specific effects depend on both the brain region analyzed (e.g., specific subregions of the striatum and BNST) and the duration of ethanol exposure (i.e., acute vs. chronic). Plastic changes in synaptic transmission in these two brain regions following prolonged ethanol exposure are thought to contribute to excessive alcohol drinking and relapse to drinking. Understanding the mechanisms underlying this plasticity may lead to new therapies for treatment of these and other aspects of alcohol use disorder.

Long-lasting changes in synaptic function (i.e., synaptic plasticity) have long been thought to contribute to information storage in the nervous system (Kandel et al. 2014; Lovinger 2010). Studies combining behavioral and physiological analyses offer strong evidence supporting this hypothesis (Kandel et al. 2014; Ramirez et al. 2014). On the one hand, this plasticity allows the organism to adapt to and function in complex environments; on the other hand, certain events or exposure to various substances can produce plasticity that has negative consequences for the organism (Ursano et al. 2009). Two main types of plasticity are long-term depression (LTD) and long-term potentiation (LTP).

Exposure to drugs of abuse, including beverage alcohol (i.e., ethanol), is one life experience that can activate or alter synaptic plasticity, often resulting in increased drug seeking and taking, and in many cases in addiction (Kauer and Malenka 2007; Luscher and Malenka 2011). Ethanol’s actions alter or produce lasting synaptic plasticity in a variety of brain regions, including two regions with key roles in alcohol’s actions as well as in control of alcohol intake, namely the striatum and the bed nucleus of the stria terminalis (BNST). These brain regions, in turn, are integral components of several brain circuits, including the cortico-basal ganglia circuits and the extended amygdala. By understanding the types of alcohol-induced synaptic plasticity in these brain regions, we can determine how the drug changes this circuitry. This information will aid in prevention or reversal of such circuit changes in the treatment of alcohol use disorder. The table summarizes the different types of plasticity discussed in this article, as well as the effects of acute and chronic ethanol exposure in these brain regions.

After a brief overview of the cortico-basal ganglia circuits and extended amygdala, this article will discuss ethanol’s effects on synaptic plasticity, focusing on the changes produced by ethanol exposure in brain regions that are prominent in the three cortico-basal ganglia circuits. One of the main foci will be on three striatal subregions that have central roles in action control by the three circuits. The article also explores ethanol–plasticity interactions in the BNST, because this brain region has emerged as a prominent player in the drive to obtain drugs of abuse, including ethanol, and also plays a major role in stress–addiction interactions and negative reinforcement.

The many mechanisms that contribute to long-lasting synaptic plasticity have been reviewed extensively in recent years (Atwood et al. 2014; Kandel et al. 2014) and therefore will not be discussed in detail in this review. Similarly, a relatively large literature has described ethanol’s effects on synaptic plasticity in other brain regions, especially the hippocampus, that contribute to ethanol-induced cognitive impairment and other aspects of intoxication and the neural effects of chronic alcohol. For example, Zorumski and colleagues (2014) have recently reviewed ethanol’s effects on synaptic plasticity throughout the brain and its relationship to altered learning and memory. Therefore, this review will focus on basic mechanisms of synaptic plasticity in BNST and striatum subregions as well as the effects of acute and chronic ethanol exposure on such plasticity. The article concludes with a discussion of the potential contribution of ethanol-induced changes in plasticity in the overall effects of this much-abused drug on the central nervous system, and the potential for interventions that may be developed based on these findings and which eventually may aid in the treatment of alcohol use disorder.

## Cortico-Basal Ganglia Loops

A conserved anatomical/physiological motif in the forebrain is the existence of at least three cortico-basal ganglia circuits known as the associative, sensorimotor, and limbic circuits, each of which represents a “loop” connecting the cortex to the basal ganglia and from there back to the cortex (Balleine and O’Doherty 2010; Yin and Knowlton 2006). These circuits help process information about sensory input and internal states as well as generate actions and sequences of actions based on that information. They include the following components:

- The associative circuit consists of associative cortices (e.g., prefrontal cortex and entorhinal cortex), the dorsomedial striatum (DMS) (which corresponds to the caudate nucleus in primates), the downstream basal ganglia subregions, and the thalamus and its projections to the cortex that complete the overall loop structure.
- The limbic circuit connects limbic cortices, including neocortical areas (e.g., medial prefrontal cortex) and “older” cortex (e.g., hippocampus and lateral amygdala), with the ventral striatum (i.e., nucleus accumbens [NAc]) and specific downstream ganglia.
- The sensorimotor circuit includes sensory and motor cortices that project to the dorsolateral striatum (DLS) (which corresponds to the putamen nucleus in primates), with particular basal ganglia and thalamic regions completing this circuit.

Although each circuit likely serves several functions within this overall context, some clearly defined subcircuit functions have emerged (Balleine and O’Doherty 2010; Yin and Knowlton 2006). The associative circuit participates in learning and performing actions based on the outcomes associated with those actions (i.e., goal-directed behavior). This circuit also seems to have a strong role in reward processing (Reynolds et al. 2001; Wickens et al. 2007). The limbic circuit not only integrates information about reward with affective state, but also appears to function prominently in determining the relationship between environmental stimuli and reward. These so-called stimulus–outcome associations contribute to Pavlovian learning and Pavlovian-instrumental transfer (Corbit and Balleine 2011). The sensorimotor circuit features prominently in control of actions by environmental stimuli and perhaps also by internal states. One characteristic of actions controlled by the sensorimotor circuit is that they become less dependent on the expected outcome of an action at any given time and instead are related to the past outcome history (Balleine and O’Doherty 2010; Yin and Knowlton 2006). These sorts of actions are often referred to as habits.

## Extended Amygdala

The extended amygdala is a group of structures that includes the amygdala proper, the BNST, and the outer part (i.e., shell) of the NAc. These regions receive input from the prefrontal cortex, thalamus, and hippocampus, usually through connections using the neurotransmitter glutamate, and project to structures in the midbrain, hindbrain, and hypothalamus. This connectivity suggests that the extended amygdala can act as a means to coordinate broad behavioral states. This anatomical construct, and in particular the central nucleus of the amygdala and the BNST, has received much attention for its role in the regulation of negative reinforcement. Briefly, dysregulation of activity in the central nucleus of the amygdala is thought to alter output to the BNST. The BNST, in turn, can then regulate stress responses by activating the body’s hormonal stress response system (i.e., the hypothalamic–pituitary–adrenal axis), as well as reward behavior by acting on regions called the ventral tegmental area and dorsal raphe nucleus. In general, the extended amygdala does not seem to directly influence functions in the dorsal striatum. However, because neurotransmitters involved in stress responses and reward behaviors (e.g., corticosterone, dopamine, and serotonin) can influence striatal plasticity, a functional link clearly exists between the extended amygdala and the striatum.

Subregions within the extended amygdala either are part of the limbic cortico-basal ganglia circuit or interact heavily with the main regions within this circuit. In this context, the BNST is of particular interest, because it not only influences striatal function but is also related to addiction and responsivity to alcohol as well as to relapse. In addition, the BNST may be part of a “neuroendocrine” or “interoceptive” basal ganglia circuit (Dong and Swanson 2003, 2004; Dong et al. 2001*a*,*b*) and therefore also fits into the general circuitry system described above.

A growing body of literature suggests the involvement of BNST in addiction. As part of the central extended amygdala this brain region is extensively interconnected with hypothalamic, midbrain, and hindbrain regions (Walker et al. 2003). In addition to the complex inputs to and outputs from the BNST, the structure itself comprises multiple subregions and cell types, the details of which are only now emerging (for a review, see Lowery-Gionta and Kash 2014). The BNST is altered, either functionally or structurally, by a variety of by a variety of drugs, including morphine, cocaine, heroin, and ethanol, and is critical for stress-related reinstatement of drug-seeking behavior (for a review, see Lowery-Gionta and Kash 2014). The BNST also is essential for alcohol–withdrawal-induced anxiety, conceptually supporting the hypothesis that the BNST regulates relapse to ethanol, and use (Huang et al. 2010). Other studies have demonstrated the involvement of the BNST in the modulation of stress- and anxiety-related behaviors (Walker et al. 2009). Given that stress and anxiety may be essential in shaping alcohol-related behavioral pathology as well as the connectivity of the BNST to mid- and hindbrain regions that can broadly influence the brain, understanding how both acute and chronic alcohol exposure can regulate plasticity in the BNST is crucial.

## Striatal Synaptic Plasticity

### LTP

Various types of activity-dependent synaptic plasticity have been observed in the dorsal and ventral striatum, including LTP and LTD. LTP is a process leading to long-lasting enhancement of signal transmission between two neurons that occurs when the two neurons are stimulated repeatedly at the same time. Similarly, LTD refers to a process by which signal transmission between two cells decreases after repeated stimulation. Both of these processes are thought to contribute to memory and learning. LTP occurs, for example, at glutamatergic synapses in the striatum. This process seems to involve mechanisms very similar to those implicated in the best- characterized LTP subtypes that occur at hippocampal synapses (Bliss and Collingridge 2013; Calabresi et al. 2007; Gerfen and Surmeier 2011). Induction of LTP in the striatum begins with activation of certain postsynaptic receptors for the neurotransmitter glutamate (i.e., the N-methyl-d-aspartate [NMDA] receptors [NMDARs]). Activation of these receptors ultimately results in an increase in transmission, most likely through the insertion of another type of glutamate receptor (i.e., alpha-amino-3-hydroxy-5-methyl-4-isoxazolepropionic acid [AMPA] receptors [AMPARs]) into the synaptic plasma membrane (Gerfen and Surmeier 2011). Striatal glutamatergic LTP is not as well characterized as is hippocampal LTP; however, it is known to have a few unique features—for example, regarding the signaling cascades (i.e., G-protein signaling) that are induced during LTP. Thus, for striatal LTP, the activation of G-protein–coupled receptors that stimulate a certain type of G-protein (i.e., the GαS type of G-protein) seems to be a key step. The majority of experiments indicate that activation of D1 dopamine receptors also is a crucial step in striatal LTP; however, these receptors are only expressed on a certain cell type (i.e., direct pathway medium spiny projection neurons [MSNs]) that make up about 45 percent of all striatal neurons (Calabresi et al. 2007). In the other striatal neurons, different classes of Gs-coupled receptors may be involved, and indeed the A2A-type adenosine receptor has been implicated in LTP induction (Gerfen and Surmeier 2011). Activation of other signaling pathways (i.e., protein kinases) also may contribute to striatal LTP.

### LTD

The major form of LTD at striatal glutamatergic synapses involves a decrease in the probability that vesicles containing the neurotransmitter fuse with the membrane of the signal-emitting (i.e., presynaptic) neuron and release their glutamate, thereby initiating signal transmission (Atwood et al. 2014). The best-characterized form of this LTD involves release by the signal-receiving (i.e., postsynaptic) cell of an endocannabinoid (eCB) that then acts back on the presynaptic cell, where it activates presynaptic CB1 receptors, resulting in inhibition of glutamate release. Induction of this form of LTD requires the activation of D2 dopamine receptors and a glutamate receptor subtype (i.e., group I mGluR receptors). Numerous other neuro-modulators also can initiate LTD at striatal synapses, including activation of certain serotonin receptors (i.e., 5-HT1b receptors) and another group of glutamate receptors (i.e., mGluR2 receptors) (Atwood et al. 2014). Despite their diversity, these receptors all couple to Gi/o-type G-proteins and produce long-lasting suppression of neurotransmitter release from the presynaptic cell.

Other forms of synaptic plasticity also have been described at glutamatergic synapses in striatum. These include NMDAR-dependent depotentiation of glutamatergic transmission onto MSNs (Calabresi et al. 2007), as well as plasticity of glutamatergic transmission onto non-MSN striatal neurons.

In addition to glutamatergic synapses, those that use γ-aminobutyric acid (GABA), the major inhibitory neurotransmitter in the brain, also exhibit both LTP and LTD (McBain and Kauer 2009; Nugent and Kauer 2008). For example, eCB-mediated presynaptic LTD has been described at GABAergic synapses onto MSNs in the striatum (Adermark and Lovinger 2009; Mathur et al. 2013). There seem to be two subtypes of eCB-dependent LTD at GABAergic synapses in this region, depending on the types of cells forming the synapses. Thus, one subtype of eCB-dependent LTD affects MSN–MSN synapses and another affects synapses transmitting signals from fast-spiking parval-bumin-expressing interneurons (FSIs) to MSNs. Activation of 5-HT1b receptors also produces LTD at striatal GABAergic synapses, and the mechanisms of this presynaptic form of LTD appear to overlap with those of eCB-mediated LTD (Mathur et al. 2011). More work is needed to determine what other forms of synaptic plasticity occur at these GABAergic synapses, because control over this inhibitory neurotransmitter presents a powerful tool to regulate striatal output, which could strongly influence action selection and responses to drugs of abuse.

## BNST Synaptic Plasticity

### LTP

Similar to the striatum, both LTP and LTD have been found in the BNST. Thus, Weitlauf and colleagues (2004) determined that extended stimulation in the dorsal lateral BNST could support LTP and that this process was dependent on NMDAR signaling. At the time, NMDAR-dependent LTP was thought to require signaling via the GluN2A receptor subunit (Liu et al. 2004), based on studies using a new compound called NVP-AAM077 that was purported to selectively block the GluN2A subunit. Consistent with previous studies, NVP-AAM077 blocked LTP in the BNST, suggesting the involvement of GluN2A (Weitlauf et al. 2005). However, both LTP and the inhibitory effect of NVP-AAM077 persisted in mice that did not carry any GluN2A receptors. Subsequent mechanistic studies found that under certain conditions NVP-AAM077 also could inhibit NMDARs containing the GluN2B receptor subunit, suggesting that the selectivity of this compound may not be as strong (Frizelle et al. 2006). More recently, Wills and colleagues (2012) used an experimental approach that could control activation and inactivation of the GluN2B subunit to demonstrate that this form of LTP is critically dependent on the presence of GluN2B-containing NMDARs.

In addition to this form of LTP, other investigators identified a novel type of LTP of intrinsic excitability that occurred in the juxtacapsular nucleus of the BNST after a high-frequency stimulation and which could be prevented by inhibition of both NMDA and mGluR5 receptors (Francesconi et al. 2009*a*,*b*). This type of plasticity may be a homeostatic mechanism to prevent excessive anxiety-like behaviors, because the juxtacapsular nucleus has been proposed to have a feedback inhibitory input to the basolateral amygdala, which in turn control fear responses.

### LTD

Several forms of LTD can be expressed in the BNST. Grueter and colleagues (2006) identified a mGluR5-mediated form of LTD induced by activation of group 1 mGluR receptors. This particular plasticity was cannabinoid independent but required extracellular signal-regulated protein kinase (ERK). Moreover, this LTD was found to affect the postsynaptic rather than presynaptic cell and involved removal of GluR2-containing AMPA receptors from the synapse (Grueter et al 2008). A similar form of plasticity is mediated by alpha1 adrenergic receptors (α1-ARs) (McElligott and Winder 2008). At first glance, these two forms of LTD appear to be the same, because they both are mediated via Gq signaling and appear to result from postsynaptic AMPA receptor trafficking. However, the α1-AR mediated LTD results from removal of GluR1 subunits rather than GluR2 subunits from the AMPA receptors (McElligott et al. 2010). Finally, other investigators identified a third type of LTD in the BNST that was dependent on mGluR5-mediated generation of the lipid signal anandamide, which then acted on postsynaptic transient receptor potential V1 (TRPV1) channels (Puente et al. 2011). Although this type of LTD was not directly compared with the LTD identified by Grueter and colleagues (2006), it is likely that they are similar.

## Acute Ethanol and Striatal Synaptic Plasticity

### Striatal Glutamatergic Synapses

**Figure 1:**
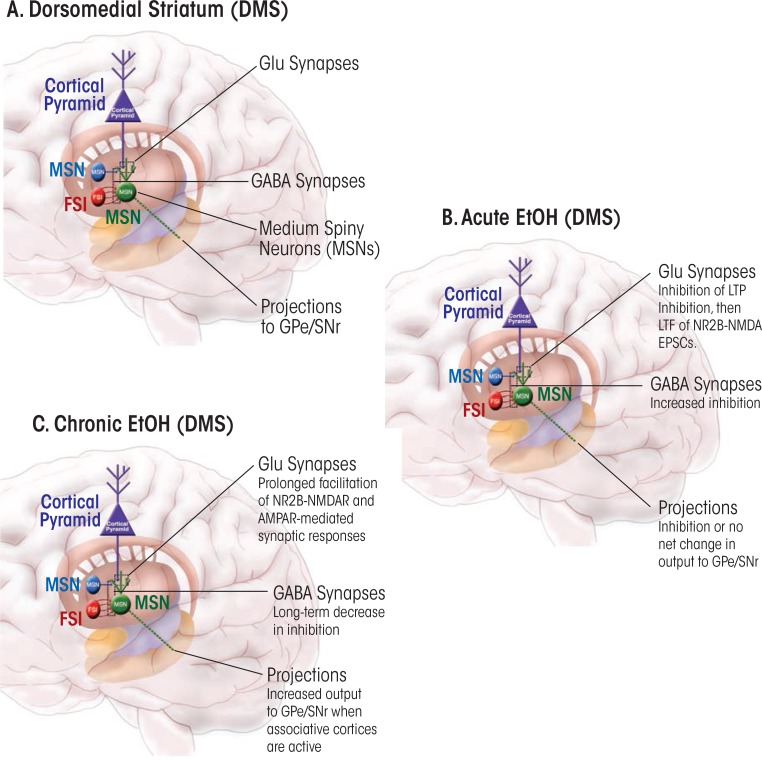
Schematic illustration of neuronal circuits in the dorsomedial striatum (DMS) and of the effects of acute and chronic ethanol exposure on plasticity in this region. **(A)** Simplified diagram of the circuits in the DMS, showing glutamatergic cortical inputs to the major projection neurons in the striatum (i.e., medium spiny neurons [MSNs]). Also indicated is GABAergic microcircuitry involving MSN–MSN synapses that tend to innervate dendrites and synapses made by fast-spiking interneurons (FSIs) on MSN cell bodies. These MSNs project out of the striatum to the globus pallidus external segement (GPe) and the substantia nigra pars reticulata (SNr). Boxed areas indicate the predominate sites of synapses on the MSNs. **(B)** Effects of acute ethanol exposure on plasticity at synapses onto DMS MSNs. The net effects are prevention of normal plasticity (i.e., inhibition of long-term potentiation [LTP]) at excitatory cortical glutamatergic inputs, while a new form of NMDA receptor (NMDAR)-dependent long-term facilitation (LTF) occurs. Increased synaptic inhibition also occurs. Thus, the net signal output from the DMS may be dampened, while responses to associative cortical input may become aberrant. **(C)** Effects of chronic ethanol exposure on plasticity at synapses in the DMS. Net effects include prolonged LTF and LTP-like increase in AMPA receptor function at glutamatergic synapses, accompanied by net decreases in inhibition. These changes may alter goal-directed ethanol-related behaviors, particularly those controlled by the prefrontal cortex and related associative cortices.

Acute intoxication occurs over a range of brain ethanol concentrations from approximately 5 to 100 mM, with increasing severity as the concentration ascends. Thus far, only a few studies have examined the acute effects of ethanol on plasticity. One series of studies using brain slices of the DMS found that a lasting facilitation of glutamatergic transmission mediated by NMDARs occurred after the slices had been exposed for a few minutes to 25 to 100 mM ethanol (Wang et al. 2007, 2010, 2012). The investigators dubbed this process long-term facilitation (LTF) to distinguish it from LTP, because LTF involves a lasting increase in NMDAR function and may not share all of the mechanisms involved in LTP. LTF occur only after the inhibition of NMDAR-mediated transmission, normally observed during acute ethanol exposure, ends (see figure 1A and 1B). It is thought that ethanol stimulates Fyn tyrosine kinase, which then mediates phosphorylation of the NR2B NMDAR subunit, thereby inducing LTF (Gibb et al. 2011; Wang et al. 2010). Indeed, LTF only affects transmission mediated by receptors that contain NR2B.

In addition to inducing LTF, acute ethanol exposure inhibits the induction of NMDAR-mediated LTP in the DMS (Yin et al. 2007) (figure 1B). Reductions in LTP magnitude, which can be observed at ethanol concentrations at the low end of the intoxicating range, most likely involve other mechanisms in addition to inhibition of NMDARs and perhaps also phosphorylation mediated by ERK. This reduction in LTP magnitude is accompanied by an increase in the magnitude of eCB-mediated LTD in DMS.

Acute ethanol exposure inhibits both LTP and LTD in the NAc/ventral striatum (Jeanes et al. 2011; Mishra et al. 2012). Thus, ethanol prevents the induction of LTP by high-frequency stimulation in this brain region (Mishra et al. 2012). This effect may involve both inhibition of responses to group I mGluRs as well as altered dopamine release. Other studies have shown that acute ethanol also alters an NMDAR-dependent form of LTD in the NAc, and particularly in the NAc shell (Jeanes et al. 2011). Normally, sustained afferent stimulation at low to moderate frequencies induces an NMDAR-dependent form of LTD in this striatal subregion (Jeanes et al. 2011; Thomas et al. 2000), which can be prevented by antagonists of NMDARs that contain the NR2B subunit. Acute ethanol exposure has a biphasic concentration-dependent effect on this NAc-LTD, with complete inhibition observed at 40 mM ethanol, but less effect at concentrations of 20 mM and 60 mM. Further analyses demonstrated that ethanol’s effect on this form of plasticity is restricted to certain cells—namely, MSNs that are part of the “direct” output pathway (Jeanes et al. 2014). In contrast to other MSNs, these cells express D1 dopamine receptors. The fact that this effect exclusively occurs in these MSNs is consistent with findings indicating that D1 receptors are involved in this form of LTD and that ethanol and D1 receptors seem to have antagonistic effects in preventing and restoring LTD (Jeanes et al. 2011). Previous work showing that D1 receptor activation counteracts ethanol-induced inhibition of NMDARs indicates a likely mechanism for this interaction.

### Striatal GABAergic Synapses

**Figure 2:**
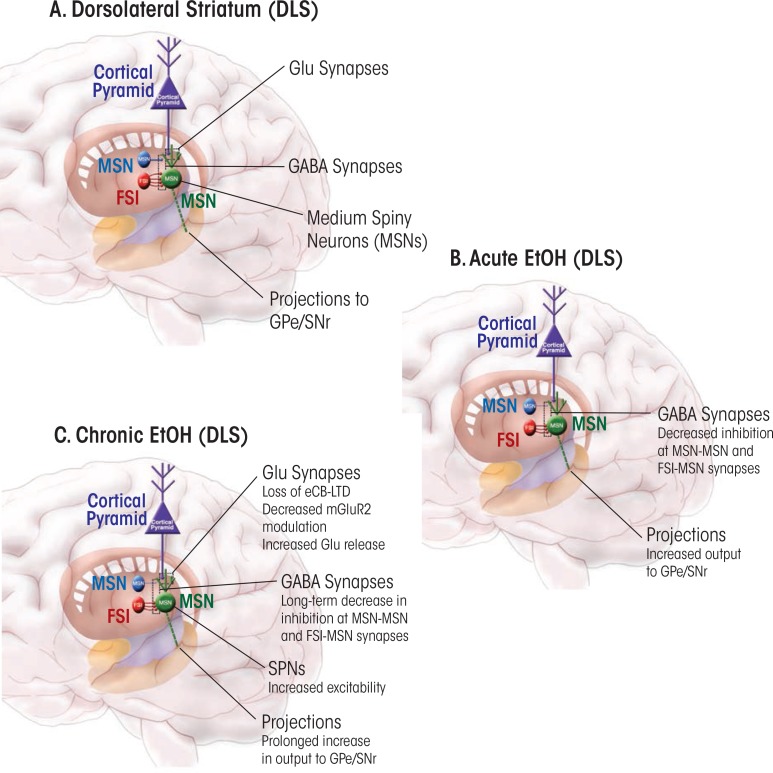
Schematic illustration of neuronal circuits in the dorsolateral striatum (DLS) and of the effects of acute and chronic ethanol exposure on plasticity in this region. **(A)** Simplified diagram of the circuits in the DLS, showing glutamatergic cortical inputs to the major projection neurons in the striatum (i.e., medium spiny neurons [MSNs]). Also indicated is GABAergic microcircuitry involving MSN–MSN synapses that tend to innervate dendrites and synapses made by fast-spiking interneurons (FSIs) on MSN cell bodies. These MSNs project out of the striatum to the globus pallidus external segement (GPe) and the substantia nigra pars reticulata (SNr). Boxed areas indicate the predominate sites of synapses on the MSNs. **(B)** Effects of acute ethanol exposure on plasticity at synapses onto DLS MSNs. The major net effect described to date is decreased inhibition, which would increase net output from sensorimotor striatum and perhaps initiate habit formation. **(C)** Effects of chronic ethanol exposure on plasticity at synapses in the DLS. The net effects are decreased presynaptic endocannabinoid (eCB)-dependent long-term depression (LTD), increased MSN excitability, and decreased inhibitory GABAergic transmission onto MSN. These changes should foster greater DLS output in response to a given set of inputs from sensorimotor cortex, potentially facilitating habit formation.

No studies have explicitly analyzed the effects of acute ethanol exposure on LTP or LTD at striatal GABAergic synapses. It is known that acute ethanol increases GABAergic transmission in the DMS, while producing the opposite effect (i.e., inhibition) at synapses in the DLS (Wilcox et al. 2014) (figure 1B and 2B). It is tempting to speculate that the increased GABA-mediated inhibition in the DMS and disinhibition in the DLS favor increased function of the sensorimotor circuit; the implications of such a scenario will be discussed later in this review.

The effects of acute ethanol exposure on GABAergic synapses in the NAc/ventral striatum also have not been examined in great detail. Nie and colleagues (2011) reported that ethanol potentiated responses to applied GABA in a subset of MSNs in the NAc core. Furthermore, Mishra and Chergui (2013) offered evidence that increased GABAergic transmission in the NAc underlies the inhibitory effects of ethanol. However, neither of these studies directly measured the effects of acute ethanol on GABA-mediated synaptic responses. A recent study indicated that acute ethanol potentiates tonic currents in the NAc that are mediated by GABA_A_ receptors (Liang et al. 2014*b*). This effect seems to be attributable to increased function of the GABA_A_ receptors, although increased GABA release also could play a role. Given the large body of literature implicating the NAc in controlling ethanol intake (Anstee et al. 2013; Hodge et al. 1995; Hyytia and Koob 1995; June et al. 1998; Nie et al. 2011; Rewal et al. 2009), additional characterization of ethanol’s effects on GABAergic transmission in this brain region certainly is warranted.

## Acute Ethanol and Synaptic Plasticity in the BNST

A few studies have examined the impact of acute alcohol on synaptic plasticity in the BNST. An initial study by Weitlauf and colleagues (2004) found that application of ethanol to a brain slice inhibited induction of LTP in the BNST. Interestingly, this impairment was limited to the early phase of LTP. Alcohol’s effect seemed to be mediated via actions on the NMDAR, because the alcohol concentration employed inhibited NMDARs but did not alter GABA_A_-mediated currents. A subsequent analysis revealed that acute alcohol specifically inhibited NMDAR-mediated but not AMPAR-mediated currents in the BNST (Kash et al. 2008*a*). In addition, this study used a pharmacological approach to determine if specific subtypes of NMDARs were involved in this inhibition. These analyses found that the presence of an NR2B-selective antagonist, Ro 25-2981, prevented ethanol’s inhibitory effect, suggesting that ethanol selectively targets NR2B-containing NMDARs in the BNST to exert its inhibitory effect on LTP. This model was supported by an elegant study demonstrating that genetic inactivation of NR2B could remove the alcohol sensitivity of NMDAR-mediated responses (Wills et al. 2012).

The lack of ethanol effects on GABAergic transmission in the BNST is intriguing, especially because this structure is similar to the central nucleus of the amygdala, where ethanol robustly increases GABA release (Gilpin et al. 2014). Furthermore, Wills and colleagues (2013) confirmed that ethanol had no effect on GABA transmission in the BNST in adult animals, but could enhance GABA transmission in adolescent animals. This developmental control of sensitivity to ethanol is interesting, because it suggests that alcohol modulation of plasticity and circuitry is dynamic. Considering the connectivity of the BNST and its role in the regulation of anxiety-like behavior and arousal states, it is tempting to speculate that ethanol inhibition of LTP in this region is linked to the anxiolytic actions of alcohol.

## Effects of Chronic Ethanol on Synaptic Plasticity at Striatal Synapses

### Striatal Glutamatergic Synapses

It has long been postulated that ethanol-induced alterations in synaptic function underlie many of the drug’s neuroadaptive effects that contribute to tolerance, physical dependence, and addiction (Lovinger and Roberto 2013; Vengeliene et al. 2008; Zorumski et al. 2014). Most research on this topic has focused on brain regions other than striatum. In recent years, however, a number of groups have begun to examine how striatal synapses are altered during and following chronic ethanol exposure. From these studies, interesting patterns are emerging that indicate how production of aberrant communication at synapses in corticostriatal circuits and striatal microcircuits contributes to alterations in responses to alcohol-associated cues, the rewarding properties of ethanol, and habitual alcohol-seeking/drinking behavior that are characteristic of alcohol use disorder.

### Effects on LTD

The eCB-dependent striatal LTD discussed earlier may be important in forms of learning and memory that involve this brain region (DePoy et al. 2013; Hilario et al. 2007; Yin et al. 2009). In particular, Hilario and colleagues (2007) found that CB1 receptors are key contributors in “habitual” learning and performance of instrumental actions (i.e., actions that are relatively insensitive to reward contingency). This type of learning involves the dorsal striatum, and in particular the sensorimotor striatum or DLS. Thus, it is possible that the promotion of habitual alcohol-seeking after repeated exposure to the drug (Barker et al. 2010; Corbit et al. 2012; Dickinson et al. 2002; Mangieri et al. 2012) involves changes in striatal eCB-mediated LTD. Indeed, Xia and colleagues (2006) found that this type of LTD decreased in magnitude in animals administered ethanol for 10 days, with the decrease persisting after ethanol withdrawal (figure 2C). Loss of eCB-LTD in the DLS also was observed in brain slices obtained from animals that had been exposed to ethanol using an inhalational model (DePoy et al. 2013). This loss was accompanied by an increase in striatal levels of an eCB called 2-arachidonoyl glycerol (2-AG). It is possible that unnaturally high levels of this eCB produce excessive LTD in the in vivo state. This in vivo LTD may occlude subsequent induction of LTD in slices. Alternatively, the high 2-AG levels could alter signaling mechanisms necessary for LTD induction in the brain slices (e.g., by causing reductions in the number of receptors or impaired signaling). It is tempting to speculate that this loss of plasticity contributes to ethanol effects that promote habitual drug seeking, but experiments to test this hypothesis have not yet been carried out.

The eCB-mediated LTD is just one form of presynaptically expressed LTD that is initiated by the activation of Gi/o-coupled receptors (Atwood et al. 2014). Glutamatergic synapses in the striatum (including those on MSNs in both dorsal and ventral striatum) appear to express presynaptic LTD driven by several receptor subtypes. For example, activation of presynaptic group II mGluRs (which includes mGluRs 2 and 3) can induce LTD in the striatum. Recent studies indicate that mGluR2 is the main receptor subtype involved in this form of LTD and that loss of this receptor is associated with higher ethanol intake in rats and mice (Meinhardt et al. 2013; Zhou et al. 2013). Indeed, in the widely studied ethanol-preferring (P) rats developed by selective breeding, the gene encoding mGluR2 receptors is defective and causes premature termination of the receptor (Zhou et al. 2013).

### Effects on LTP

LTP also is thought to have key roles in learning and memory in a variety of brain regions (Kandel et al. 2014), including the striatum (Dang et al. 2006; Lovinger 2010; Reynolds et al. 2001). Chronic exposure to drugs of abuse may alter or engage pivotal synaptic plasticity mechanisms to produce learning and memories related to the drugs and associated environmental events. Thus, the effects of drugs on LTP have been widely studied, and it is clear that chronic ethanol exposure alters LTP in brain regions such as the hippocampus (for a review, see Zorumski et al. 2014).

In the striatum, the net effect of chronic ethanol has been less well studied, but the work to date indicates that ethanol exposure brings about an LTP-like enhancement of synaptic efficacy at glutamatergic synapses that transmit signals to striatal MSNs. As mentioned previously, Wang and colleagues (2010) have described an increase in NMDAR-mediated synaptic transmission that begins just after the end of an acute exposure to ethanol and which seems to be largest in the DMS (figure 1C). Because the AMPAR-mediated component of glutamatergic transmission is unaltered at that stage, the observed LTF seems to be specific to NMDARs (Wang et al. 2010). However, given the key role of NMDARs in the LTP induction process, increasing NMDAR function might be expected to increase the likelihood of induction of an LTP-like form of plasticity that would involve increased AMPAR-mediated transmission (Collingridge 2003). Indeed, further studies demonstrated that either repeated acute exposure of brain slices to ethanol or repeated in vivo ethanol exposure enhanced the ability to induce LTP in the DMS (Wang et al. 2010). Furthermore, chronic ethanol exposure enhanced not only NMDAR-mediated transmission onto DMS MSNs (Wang et al. 2010) but also synaptic AMPAR expression (Wang et al. 2012). Thus, chronic exposure to ethanol gradually sets up conditions that favor induction of LTP-like increases in glutamatergic efficacy in striatum, perhaps contributing to alterations in ethanol intake or environmental control of intake.

Indeed, LTF of NMDARs and the potentiation of AMPAR-mediated transmission both seem to contribute to altered ethanol intake. Thus, injection into the DMS (but not into the DLS) of an antagonist for NR2B-containing NMDARs reduced operant responding for ethanol (Wang et al. 2010). Antagonist injection into the DMS also decreased ethanol-primed increases in operant responding for the drug. Similarly, injection of AMPAR antagonists into the DMS decreased operant responding for ethanol (Wang et al. 2012). Further analyses have implicated a signaling pathway involving changes in phosphorylation of the NR2B subunit that involves the Fyn tyrosine kinase (Gibb et al. 2011; Wang et al. 2010). Additional evidence suggests that manipulations of the activity of this kinase as well as of protein tyrosine phosphatase α (PTPα) in the DMS alter ethanol drinking and preference (Ben Hamida et al. 2013).

### Effects on LTD and LTP in the NAc

Effects of chronic ethanol exposure on LTD and LTP of glutamatergic transmission have also been examined in the NAc shell of mice (Jeanes et al. 2011). The NAc LTD described earlier, which depended on the NR2B receptor subunit, disappears if plasticity is assessed 1 day after a relatively short inhalational exposure to chronic ethanol; instead, this stimulation protocol induces an NMDAR-dependent form of LTP. The NR2B receptor subunit is altered by both acute and chronic ethanol exposure (Carpenter-Hyland et al. 2004; Floyd et al. 2003; Wang et al. 2010, 2012), suggesting that changes in the expression or function of this subunit contribute to ethanol-induced changes in plasticity. Indeed, increased expression of the NR2B subunit occurs in the NAc following chronic ethanol exposure (Obara et al. 2009; Szumlinski et al. 2008). The decrease in LTD induced by chronic ethanol persists for 3 days, but is fully reversed 2 weeks after the end of ethanol exposure. The ethanol-exposure regimen that altered NAc-LTD also produced increased ethanol intake in mice. Thus, it is tempting to think that the changes in synaptic efficacy at NAc-shell synapses contribute to the change in intake; however, more work will be needed to confirm this hypothesis.

Other investigators recently also demonstrated decreased LTD in the NAc shell during withdrawal following chronic ethanol exposure in rats (Spiga et al. 2014). Alterations in expression of several proteins (i.e., tyrosine hydroxylase, PSD-95 postsynaptic density protein), alterations in dendritic spine morphology, and decreased NMDAR-mediated synaptic transmission all accompanied the loss of LTD. Thus, the array of molecular changes accompanying and possibly contributing to chronic ethanol-induced loss of NAc-shell LTD is expanding.

Decreased NMDAR-dependent LTD also has been observed in the NAc-core following chronic ethanol exposure and is associated with locomotor sensitization (Abrahao et al. 2013). Indeed, LTD was normal in mice that did not exhibit sensitization following repeated ethanol injections, but decreased in magnitude in those animals that exhibited sensitization following the chronic drug exposure regimen. The loss of NAc-core LTD likely was caused by decreased NMDAR-mediated synaptic responses, which accompanied this form of plasticity. It is interesting to note that mice that showed sensitization also exhibited increased ethanol intake in a drinking-in-the-dark paradigm. Thus, these findings reinforce the idea that loss of NAc LTD is associated with increased ethanol intake.

Another intriguing study has linked NMDARs with the development of ethanol drinking that does not decrease (as occurs normally) when it results in aversive consequences. Specifically, this aversion-resistant drinking was associated with increased expression of NMDARs that contain the NR2C subunit at synapses where glutamatergic cells from the medial prefrontal cortex and insular cortex connect with NAc MSNs (Seif et al. 2013). Transmission mediated by these receptors is more prominent at hyperpolarizing potentials compared with transmission mediated by receptors containing other NR2-type subunits. Furthermore, inhibition of the NR2C-containing receptors in the NAc core reduced the aversion-resistant drinking. This finding indicates that an increased contribution of NMDARs to synaptic transmission may help to drive drinking under stressful/aversive conditions via actions in the limbic circuitry.

However, it is not only changes in postsynaptic NMDAR-mediated synaptic transmission in the NAc that seem to contribute to chronic ethanol effects and alcohol-related behaviors; alterations in AMPAR-mediated synaptic transmission also seem to play a role. Thus, prolonged chronic ethanol exposure was associated with an increase in AMPAR-mediated transmission (Marty and Spigelman 2012). This increase resulted from enhancement of synaptic AMPARs lacking the GluA2 subunit, a change that has also been observed following LTP induction (Isaac et al. 2007), reinforcing the idea that prolonged ethanol exposure can induce LTP-like increases in glutamatergic transmission.

### Effects on Extracellular Glutamate

Chronic ethanol exposure also may affect extracellular glutamate levels in various brain regions, including the NAc (Gass et al. 2011; Melendez et al. 2005; Szumlinski et al. 2007, 2008). However, it is not clear if these changes involve altered synaptic glutamate release or result from increased glutamate coming from neuronal transporters or other cellular sources (for a review, see Marty and Spigelman 2012). Interestingly, prolonged chronic-intermittent ethanol exposure is associated with decreased expression of presynaptic mGluR2 by neurons in the infralimbic cortex, a prominent region in the limbic circuit. These infralimbic cortex neurons project to the NAc where they release glutamate from their presynaptic terminals. The mGluR2 on these terminals helps control glutamate levels by limiting glutamate release through autoreceptor feedback (Meinhardt et al. 2013). Accordingly, decreased function of this receptor could contribute to the increased extracellular glutamate levels associated with elevated intake following chronic exposure. Other studies demonstrated that restoring receptor expression in the infralimbic cortex reduced the elevated drinking observed in the chronically exposed animals (Meinhardt et al. 2013). These findings, along with studies showing that alcohol-preferring P rats lack mGluR2 (Zhou et al. 2013), indicate that a decrease or loss of this receptor leads to insufficient control of glutamate release, which ultimately may contribute to excessive alcohol drinking.

### Striatal GABAergic Synapses

In addition to the documented alterations in glutamatergic transmission induced by chronic alcohol, evidence indicates that chronic ethanol exposure also induces changes in GABAergic synaptic transmission in striatal micro-circuits. These forms of plasticity also may contribute to increased ethanol seeking and intake.

In the dorsal striatum, GABAergic transmission is decreased following chronic ethanol drinking. For example, GABAergic transmission declined in both the DMS and DLS of mice who had been consuming ethanol for 6 weeks under a drinking-in-the-dark regimen (Wilcox et al. 2014) (figure 1C and 2C). A similar decrease in GABAergic transmission was observed in the putamen of Cynomolgus macaque monkeys, which is roughly equivalent to mouse DLS (Cuzon Carlson et al. 2011). The reasons underlying this decrease in transmission remain to be determined. Analyses of miniature inhibitory postsynaptic currents (mIPSCs) in striatal neurons from these mice and monkeys suggested possible synaptic loci underlying the chronic ethanol-induced decrease in GABAergic transmission. The most consistent finding was a decrease in mIPSC frequency, indicating either a decrease in GABA release or a decreased number of GABAergic synapses on MSNs in the sensorimotor striatum.

Liang and colleagues (2014*a*) have examined the effects of prolonged intragastric ethanol exposure on the properties and pharmacology of GABAergic synapses and tonic GABA_A_ receptor-mediated transmission onto NAc MSNs. The major changes were in receptor pharmacology, with decreased potentiation in response to acute ethanol and diazepam and increased effects of a compound called RO15-4513. This compound partially inhibits the receptor through an action known as partial inverse agonism. These changes certainly could contribute to tolerance to the CNS effects of both ethanol and sedative benzodiazepines. Chronic ethanol also decreased the amplitude and frequency while increasing the rise time of GABAergic mIPSCs, indicating postsynaptic changes at GABAergic synapses. Furthermore, the NAcs of mice chronically exposed to ethanol exhibited changes in cell surface levels of several GABA_A_ receptor subunits, including decreased expression of alpha1 and delta subunits and increased expression of alpha4 and alpha5 subunits. Some of these changes may well contribute to the postsynaptic changes at GABAergic synapses. Surprisingly, the tonic GABA_A_ receptor-mediated current was not altered in these neurons, despite the decreased expression of the delta receptor subunit that mediates this current. Similarly, chronic ethanol exposure did not alter dopamine modulation of the tonic GABA_A_ receptor- mediated current (Liang et al. 2014*b*). Finally, chronic ethanol exposure led to decreased frequency of mIPSCs, which seemed to result mainly from a decrease in occurrence of mIPSCs with fast rise times. This may reflect a decrease in GABA release or in the number of synapses at a particular input to these MSNs.

### Striatal Synaptic Plasticity and Alcohol Seeking/Intake

When evaluating alcohol-induced changes in synaptic plasticity and alcohol-related behaviors, it is important to consider the pattern and duration of ethanol exposure. Most of the chronic exposure paradigms discussed here involve periods of ethanol availability alternating with periods of forced withdrawal or abstinence. Synaptic and behavioral changes brought about by ethanol exposure alone should be carefully compared with changes only observed when exposure and withdrawal/abstinence occur, because the in vivo outcomes differ with these different paradigms (Becker and Hale 1993; Lopez and Becker 2005).

The contributions of the various types of ethanol-induced striatal synaptic changes to the neuroadaptation and behavioral changes associated with excessive alcohol intake and alcohol dependence are the subject of considerable ongoing investigation. A prominent role for the dorsal striatum in the control of alcohol intake is just starting to emerge. Thus, it appears that the associative striatum contributes to alcohol seeking and intake at stages where these behaviors still are under the control of “goal-directed” strategies. However, when seeking and taking become more habitual (i.e., less dependent on the outcome following a behavior), the contributions of sensorimotor striatal regions may become more prominent. Although this scenario is supported by behavioral evidence, little is known about the molecular, synaptic, and cellular mechanisms that contribute to the different alcohol-seeking and -taking strategies. It will be interesting to determine how the mechanisms described above contribute to goal-directed and habitual alcohol seeking and drinking.

By contrast, many studies have critically implicated the NAc in controlling intake of alcohol and other drugs of abuse (Belin et al. 2009; Koob 2013; Marty and Spigelman 2012). The ethanol-induced synaptic alterations and changes in synaptic plasticity in that brain region have been postulated to contribute to the excessive alcohol intake associated with alcohol use disorder. Strong tests of this hypothesis have yet to be carried out. However, several studies already have implicated GABA_A_ receptors and GABAergic transmission in the control of alcohol seeking and drinking (Anstee et al. 2013; Hodge et al. 1995; Hyytia and Koob 1995; June et al. 1998; Nie et al. 2011; Rewal et al. 2009). Thus, it is very likely that the effects of chronic exposure on synaptic plasticity in the NAc play a significant part in dependence and escalated drinking following such exposure.

Ultimately, each of the three striatal subregions—DMS, DLS, and NAc—contribute to the neural and behavioral changes brought about by chronic ethanol exposure. A simple model suggests that early in our experiences with alcohol, brain regions that are sensitive to the proximal relationship between actions and reward, such as the DMS and NAc, may exert strong control over alcohol seeking and drinking. With continued ethanol exposure, both internal and environmental stimuli may begin to exert greater control over alcohol seeking and drinking by strengthening brain activity in the sensorimotor and limbic circuits that are responsive to complex stimuli and predictive cues, respectively. Furthermore, relapse induced by exposure to ethanol-associated cues appears to involve the NAc and the rest of the limbic circuit and their functions in Pavlovian-instrumental transfer (Belin et al. 2009; Corbit and Janak 2007). The limbic circuit also is strongly engaged during withdrawal and abstinence from alcohol use and thus may contribute to relapse driven by the negative consequences of such abstinence (Koob 2013). The internal states and/or external stimuli that engage the sensorimotor circuitry may not only contribute to relapse, but likely also contribute to excessive ethanol intake once relapse has occurred. Indeed, once drinking has begun, the combination of the particular context and the effects of ethanol itself may drive continued intake until significant environmental or physiological events (e.g., loss of consciousness) interfere with the habitual behavioral pattern. Future research will no doubt focus on how the different synaptic mechanisms and different brain circuits contribute to relapse and excessive drinking. Ultimately, research should strive to find ways to disrupt both processes through targeted alterations in the activity of the plasticity of the involved circuits.

## Chronic Ethanol and Synaptic Plasticity in the BNST

Given the important role that the BNST plays in regulating negative affective states and negative reinforcement, several studies have examined the ability of chronic ethanol exposure to alter synaptic plasticity in this brain region. In the initial study examining chronic alcohol exposure on BNST function, and more specifically NMDAR function, Kash and colleagues (2009) found that 4 days of chronic-intermittent, but not continuous, exposure to ethanol vapor led to a functional upregulation of NR2B-containing NMDARs. The investigators also explored the temporal summation of NMDARs in response to repeated stimulation across a range of frequencies. Changes in this summation had been suggested to be an index of metaplasticity and to reflect the potential of a circuit to induce plasticity. The study found that this summation increased across all frequencies tested (Kash et al. 2009). Based on this increase in NR2B expression, the investigators hypothesized that the acute actions of ethanol on the NMDAR response (i.e., inhibition of NMDAR-mediated currents) in the BNST would be enhanced with chronic exposure. However, ethanol inhibition of NMDAR-mediated responses actually decreased after chronic exposure. This suggests that although subunit configuration may affect regulation of alcohol responsivity, other factors also are involved, consistent with the findings by other researchers (Jin and Woodward 2006; Woodward 2000; Xu and Woodward 2006; Xu et al. 2008).

Other investigators followed up on this study by examining how chronic-intermittent ethanol-vapor exposure could specifically alter plasticity. Wills and colleagues (2012) found that two cycles of chronic-intermittent ethanol exposure led to increased LTP, consistent with the model proposed in previous studies. Moreover, NR2B-containing NMDARs seemed to be upregulated at extrasynaptic sites that seemed to be coupled to LTP. These findings were in contrast to observations in the hippocampus, suggesting that novel protein signaling complexes may be associated with NMDARs in the BNST compared with other regions. Interestingly, Conrad and Winder (2011) found that adolescent ethanol exposure, when combined with exposure to stress, led to alterations in both anxiety-like behavior and LTP in the BNST, providing further support that a functional link exists between these two measures.

Silberman and colleagues (2013) found that chronic-intermittent ethanol also led to alterations in glutamatergic function in the BNST that depended on another receptor, corticotrophin-releasing factor receptor type 1 (CRFR1). This observation is consistent with studies demonstrating that CRFR1 activation could enhance glutamate release in slice preparations (Kash et al. 2008*b*; Nobis et al. 2011).

Francesconi and colleagues (2009*a*,*b*) also investigated the impact of chronic ethanol exposure on plasticity, specifically on the LTP of intrinsic excitability in the juxtacapsular nucleus of the BNST. As mentioned previously, this is an anatomically distinct region in the BNST, reflective of a unique set of inputs and outputs (for a review, see Lowery-Gionta and Kash 2014). In contrast to other investigators (Wills et al. 2012), Francesconi and colleagues (2009*a*,*b*) found that chronic alcohol exposure led to a dampening of LTP in this BNST region. Additional analyses demonstrated that the dampened LTP resulted from an upregulation of certain potassium currents (i.e., D-type potassium currents). Similar alterations in plasticity occurred after cocaine and heroin self-administration, suggesting that this may be a common adaption to chronic exposure to drugs of abuse. Finally, the chronic ethanol- induced LTP dampening was blocked by agonists to CRFR1, providing a link between the CRF systems and altered plasticity. One potential model that takes all of these changes into account posits that chronic alcohol exposure leads to increased levels of dopamine or norepinephrine in the BNST during a behavioral challenge. The increased levels of these monoamine neurotransmitters then can activate CRFR1 signaling, potentially via depolarization of CRF neurons. The resulting increase in glutamate release can act in concert with the upregulation of NR2B to lead to increased plasticity in the BNST, potentially resulting in enhanced anxiety-like behavior.

Other studies have investigated how chronic alcohol alters LTD in the BNST. Because norepinephrine is thought to play an important role in stress-induced relapse and anxiety, McElligott and colleagues (2010) investigated how chronic-intermittent ethanol altered alpha1A receptor-mediated LTD in the BNST. The investigators found that 4 days of chronic-intermittent exposure (i.e., the same exposure regimen that enhanced NMDAR function) led to a partial loss of this LTD. In contrast, 10 days of restraint stress resulted in a total loss of LTD. Reasoning that this might result from prior induction of the LTD in vivo, which occludes the induction of LTD in slices, the investigators evaluated the presence of GluR1-lacking AMPARs in the BNST. The analyses found significant downregulation of these receptors after stress, indicating in vivo induction of this form of LTD. In contrast, cocaine exposure, which alters mGluR5-LTD, did not have any effect on this alpha1A receptor-mediated LTD. These findings support the idea that norepinephrine is released in the BNST during both stress and alcohol exposure, providing a mechanism by which the alpha1A receptor antagonist prazosin can reduce drinking and anxiety in people with alcohol use disorder (Simpson et al. 2009).

Several other studies also have examined how exposure to either drugs of abuse or stress can alter plasticity in the BNST. One series of studies focused on examining the impact of stress and nicotine exposure on CB1R-mediated plasticity. These analyses found that either stress or nicotine self-administration could reverse this LTD to an LTP (Jalabert et al. 2009; Massi et al. 2008; Puente et al. 2010; Reisiger et al. 2014). The mechanism underlying this switch in the polarity of plasticity is unclear at this point. One might hypothesize that because alcohol exposure and withdrawal are stressful, they would lead to similar changes in function; however, this has yet to be determined. Other researchers demonstrated that cocaine self-administration led to a novel LTP of GABA transmission mediated via neurotensin signaling (Krawczyk et al. 2013). Together with previous observations that cocaine can lead to CRF-dependent changes in glutamatergic transmission (Kash et al. 2008*b*; Nobis et al. 2011), this finding suggests that neuropeptide signaling may have an essential function in the regulation of plasticity in the BNST.

In summary, all of these findings indicate that alcohol can affect plasticity in the BNST; however, the specific effects likely are dependent on the subregion of the BNST and potentially even the neuronal subtype being targeted. Future work using viruses and reporter mice to specifically target molecules potentially involved in these processes will clarify and extend these results. Moreover, it is possible to move these studies beyond correlational analyses and determine the effect that these various forms of plasticity have on alcohol-related behaviors, using in vivo optogenetic^1^ and chemical genetic methods. This will be essential in understanding how to target and treat discrete aspects of alcohol addiction.

## Summary

Both acute and chronic ethanol exposure can modulate synaptic function and plasticity in the dorsal striatum and the BNST. Both of these regions seem to play important but distinct roles in alcohol-related behavioral plasticity. A challenge for the entire alcohol research field will be defining the molecular targets and mechanisms that mediate these ethanol-induced changes in function. It will also be critical to move beyond correlational studies and begin to define how these changes in circuits can directly regulate behavior. Elucidation of the pathways linking changes in brain plasticity to behavior hopefully also will point out potential new targets for the amelioration, reversal, or prevention of alcohol-induced changes in brain circuitry. The identification of such targets could open new avenues for translational research into novel or more effective treatment of alcohol use disorder.

## Figures and Tables

**Table:** Synaptic Plasticity and Effects of Acute and Chronic Ethanol on Long-Term Potentiation (LTP) and Long-Term Depression (LTD) in the Dorsolateral Striatum (DLS), Dorsomedial Striatum (DMS), Nucleus Accumbens (NAc), and Bed Nucleus of the Stria Terminalis (BNST)

	**LTP**	**LTD**	**Acute Ethanol**		**Chronic Ethanol**	
	
			**LTP**	**LTD**	**LTP**	**LTD**
DMS/DLS Glutamatergic Synapses	**DMS/DLS** Activation of N-methyl-d-aspartate (NMDA) receptors (NMDARs) Insertion of alpha-amino-3-hydroxy-5-methyl-4-isoxazolepropionic acid (AMPA) receptors (AMPARs) Stimulation of alpha subunit of the Gs type G protein (Gαs) signaling Involvement of A2A-type adenosine receptors Involvement of protein kinase signaling	**DLS** Decreased probability of vesicle fusion, glutamate release Endocannabinoid (eCB- mediated inhibition of glutamate release Activation of dopamine receptor type 2 (D2) dopamine receptors Activation of metabotropic glutamate receptor (mGluRs) (groups I and II) Stimulation of Gi/Go G proteins	**DMS** Long-term facilitation of glutamatergic transmission Involves inhibition of NMDAR transmission Involves stimulation of Fyn tyrosine kinase (TK), phosphorylation of NR2B Inhibition of NMDAR mediated LTP	**DMS** Increase in eCB-mediated LTD	**DMS** Increased LTP Involves NMDARs (NR2B) and AMPARs Involves Fyn TK and protein tyrosine phosphatase alpha (PTPα)	**DLS** Decreased eCB LTD Secondary to increased 2-AG levels Loss of mGluR2
DMS/DLS GABAergic Synapses	Unknown	**DLS** eCB-mediated LTD at medium spiny neuron (MSN)–MSN and MSN–fast-spiking interneuron (FSI) synapses Activation of serotonin receptor type 1B (5HT1b) receptors	Unknown (increased γ-aminobutyric acid [GABA] release in DMS and decreased GABA release in DLS)	Unknown	Unknown	Decreased GABA release in DMS and DLS
NAc Glutamatergic Synapses	NMDAR activation AMPAR insertion	NMDAR-dependent mechanisms (NR2B) AMPAR removal	Decreased LTP Dependent on mGluR (group 1) Involves altered dopamine release Decreased LTD Biphasic, concentration-dependent effect	Decreased LTD Restricted to direct pathway MSNs Involves dopamine receptor type 1 (D1) receptor	Increased AMPAR function (similar to LTP)	Decreased LTD in both shell and core Increased NR2B Persists for 3 days in shell, recovers after 2 weeks Decreased tyrosine hydroxylase (TH) and postsynaptic density 95 protein (PSD-95) in shell Decreased extracellular GluR2
NAc GABAergic Synapses	Unknown	Unknown	Unknown (increased GABAergic transmission)	Unknown	Unknown	Decreased GABA release, altered GABAAR pharmacology & decreased α1 and δ subunits
BNST Glutamatergic Synapses	Activation of NMDA receptors (NR2A and NR2B subunits) LTP subtype mediated by NMDA and mGluR5 receptors	Dependent on mGluR5, extracellular signal–regulated kinase (ERK) Involves removal of GluR2 AMPARs from synapse Mediated by α1 adrenergic receptors Requires Gq signaling Due to removal of GluR1 AMPAR from synapse Involves anandamide and transient receptor potential vanilloid 1 (TRPV1) channels	Inhibition of LTP Mediated by NMDAR (NR2B)	Unknown	Increased Increased NR2B Dampening of LTP in juxtacapsular nucleus	Decreased Downregulation of α1A AMPAR

## Footnotes

1 Optogenetic approaches use light to control the activity of neurons that have been genetically modified so that they become responsive to light.
